# Pupil Dilation: A Fingerprint of Temporal Selection During the “Attentional Blink”

**DOI:** 10.3389/fpsyg.2012.00316

**Published:** 2012-08-28

**Authors:** Ariel Zylberberg, Manuel Oliva, Mariano Sigman

**Affiliations:** ^1^Laboratory of Integrative Neuroscience, Physics Department, FCEyN UBA and IFIBA, ConicetBuenos Aires, Argentina; ^2^Instituto de Ingeniería Biomédica, Facultad de Ingeniería, Universidad de Buenos AiresBuenos Aires, Argentina; ^3^Department of Vision and Cognition, Netherlands Institute for Neuroscience, an Institute of the Royal Netherlands Academy of Arts and SciencesAmsterdam, Netherlands

**Keywords:** attentional blink, pupil dilation, timing of attention, psychological refractory period, processing bottleneck

## Abstract

Pupil dilation indexes cognitive events of behavioral relevance, like the storage of information to memory and the deployment of attention. Yet, given the slow temporal response of the pupil dilation, it is not known from previous studies whether the pupil can index cognitive events in the short time scale of ∼100 ms. Here we measured the size of the pupil in the Attentional Blink (AB) experiment, a classic demonstration of attentional limitations in processing rapidly presented stimuli. In the AB, two targets embedded in a sequence have to be reported and the second stimulus is often missed if presented between 200 and 500 ms after the first. We show that pupil dilation can be used as a marker of cognitive processing in AB, revealing both the timing and amount of cognitive processing. Specifically, we found that in the time range where the AB is known to occur: (i) the pupil dilation was delayed, mimicking the pattern of response times in the Psychological Refractory Period (PRP) paradigm, (ii) the amplitude of the pupil was reduced relative to that of larger lags, even for correctly identified targets, and (iii) the amplitude of the pupil was smaller for missed than for correctly reported targets. These results support two-stage theories of the Attentional Blink where a second processing stage is delayed inside the interference regime, and indicate that the pupil dilation can be used as a marker of cognitive processing in the time scale of ∼100 ms. Furthermore, given the known relation between the pupil dilation and the activity of the locus coeruleus, our results also support theories that link the serial stage to the action of a specific neuromodulator, norepinephrine.

## Introduction

When two masked stimuli are presented in close succession, identification of the first one (T_1_) hinders the detection of the second (T_2_) if both are presented within 200–500 ms (Raymond et al., 1992). This observation, referred as the Attentional Blink (AB), has been described in terms of two-stage theories of conscious access (Chun and Potter, 1995), where an initial effortless feed-forward propagation of stimulus information is followed by a second wave of stimulus amplification incorporating contextual and task-related information (Dehaene et al., 2006). The second wave has been linked to the action of selective attention (Dehaene et al., 2003). When two targets are presented in close succession, attention cannot be directed to T_2_ due to capacity limits (Marois and Ivanoff, 2005) or strategic control (Taatgen et al., 2009), and T_2_ is often missed (Bowman and Wyble, 2007; Zylberberg et al., 2009).

Several theories of attention and consciousness have argued that the AB is a close sibling to other observation in psychology, the Psychological Refractory Period (PRP). In the PRP, two stimuli requiring speeded responses are presented in close succession and the response to the second one is postponed (Pashler, 1994; Sigman and Dehaene, 2005). The PRP and the AB are related phenomena which can even be obtained within the same paradigm (Wong, 2002). Mixed AB-PRP experiments have shown that response speed to T_1_ can modulate the duration of the AB (Jolicoeur, 1998, 1999), and that blinked and PRP trials generate similar brain activations at the early sensory levels that diverge for late activations (>350 ms) in frontal areas, which are delayed in the PRP and absent on blinked trials (Marti et al., 2011). The link between the blink and the PRP is also supported by modeling studies showing that many AB findings can be explained by multitasking models developed for the PRP and related phenomena (Taatgen et al., 2009; Zylberberg et al., 2010).

Previous work attributed the interference observed in the AB to the action of a specific neuromodulator, norepinephrine (Nieuwenhuis et al., 2005). The locus coeruleus (LC) is the most important norepinephrine (NE) nucleus in the brain, with wide projections to most of the neocortex (Feldman et al., 1997). The LC neurons fire a burst of activity following behaviorally relevant sensory events (Aston-Jones and Bloom, 1981), which produces an excitatory effect on the cortex but an inhibitory effect on the LC itself. Thus, after this burst of activity LC-NE neurons show a refractory period of up to a few hundred milliseconds during which the activity of the LC is suppressed. This refractory-like period may be responsible for the performance deficit observed in the AB (Nieuwenhuis et al., 2005). Accordingly, the LC appears to play an important role in the generation of the P300 component (Berridge et al., 1993), a strong deflection observed in the scalp EEG which is associated with attentional mechanisms and is strongly modulated by the AB (Sergent et al., 2005). While measuring LC activity in humans is not generally feasible, pupil dilatation can be used as an indirect measure of LC activity (Gilzenrat et al., 2010). Moreover, a recent study has shown that even if fluctuations in pupil size are filtered by slow temporal signals, pupil dilatation can convey information with high temporal precision which can be recovered with deconvolution techniques (Wierda et al., 2012). This methodology resembles techniques which can improve the temporal resolution of fMRI factoring out the filtering with the slow hemodynamic function (Menon et al., 1998; Sigman et al., 2007).

Here we investigate the time course of the cognitive signals indexed by the pupil dilation in the fast time scale of ∼100 ms. Our hypothesis is that the pupil dilatation should reveal a PRP-type profile, suggesting LC driven episodes of attention (Bowman and Wyble, 2007) which cannot be identified by behavioral observation alone.

## Results

**Figure 1:**
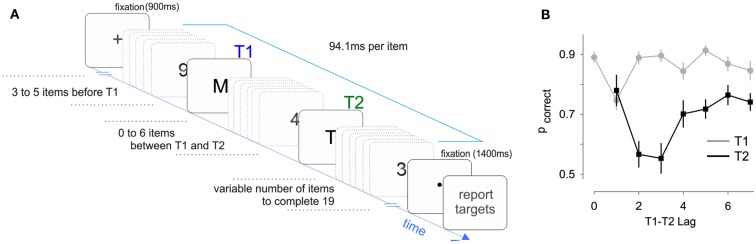
**Task design and performance**. **(A)** Sketch of the experiment. Subjects had to report the identity of the letters embedded within a sequence of distracting numbers. Subjects were required to keep fixation both before and after the RVSP. **(B)** Accuracy for the first and second targets revealed a classic “Attentional Blink” effect, with T_2_ accuracy reduced at lags 2 and 3. Lag 0 corresponds to the presentation of a single-target. Accuracy for T_2_ was only evaluated on trials where T_1_ was correctly reported.

Fifteen participants performed an AB experiment where two targets had to be identified within a RSVP (rapid serial visual presentation) of distracting stimuli (Figure 1A). The main experimental variable in the AB is the lag between the two targets, which in our experiment was made to vary between 1 (T_2_ presented immediately after T_1_) and 7.

T_2_ performance as a function of lag revealed the distinctive AB pattern. The lag between T_1_ and T_2_ had a significant effect on T_2_ performance, which we tested with a nested regression comparing models with and without lag as independent variable (*F*_6,3773_ = 24.7, *p* < 10^−8^). We also observed a clear “lag-1 sparing” (Martin and Shapiro, 2008), whereby accuracy is higher at lag-1 than at lags 2 and 3 (Figure 1B). Overall, these results indicate that our paradigm produced a classic AB effect, which allowed us to investigate how the pupil varies with performance and lag.

**Figure 2:**
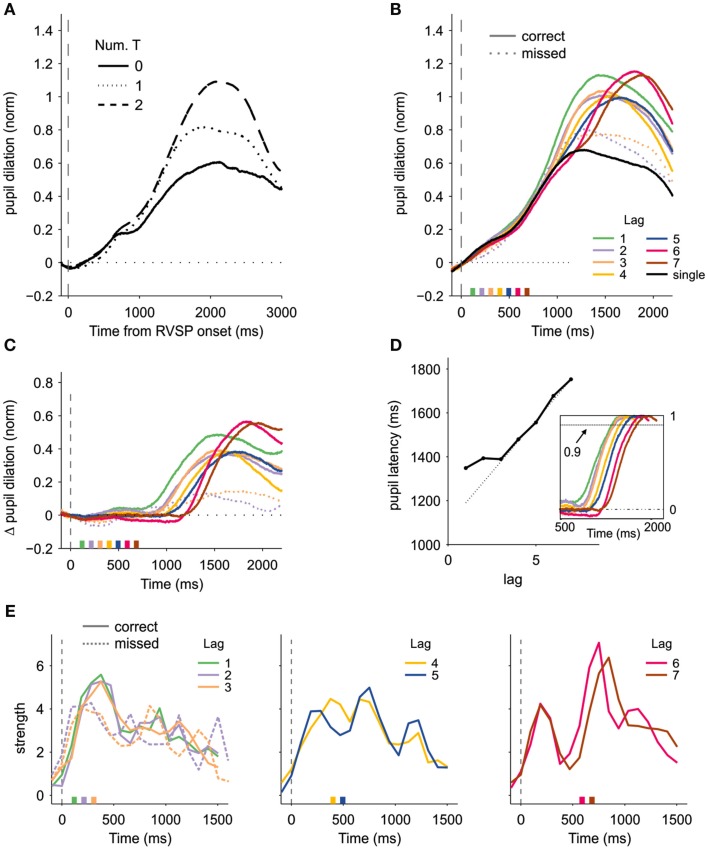
**Pupil dilation and the timing of attention**. **(A)** Normalized pupil dilation as a function of the number of reported targets, locked to the onset of the RVSP. **(B)** Pupillary response as a function of lag and accuracy, aligned to the onset of the first target. The dashed line shows the time course of the pupil dilation for missed T_2_, for lags 2 and 3. The solid black line shows the response when only one target was presented. The boxes at the bottom of the graph indicate the onset of the second target. **(C)** Same as in **(B)**, after subtracting the pupil size when only one target had to be reported. **(D)** Latency of the pupil response (arbitrarily defined as the point in the where the dilation reached 90% of its peak) as a function of lag, showing that the pupil dilation is protracted at short lags. Pupil latencies are shown relative to the onset of T_2_. The dashed line indicates the expected latency if the pupillary responses were time-locked to T_2_: a good predictor of the actual latency only at long lags. The inset shows the pupillary response [as in **(C)**] divided by the peak response at each lag. **(E)** Strength of the attentional pulses obtained after deconvolving the pupillary response with an impulse response function following the method introduced in (Wierda et al., 2012). Solid lines correspond to correct trials, and dashed lines to blinked trials (only for lags 2 and 3).

Pupil size (arbitrary units) was normalized for each trial by subtracting the baseline (computed over a temporal window between −300 and 300 ms relative to the onset of the RVSP) and dividing by its standard deviation across trials. First, we averaged the size of the pupil according to the number of reported targets (Figure 2A). We found that pupil dilation increased with the number of responses, showing a larger dilation for two than for one target (*p* < 0.5 × 10^−3^, one sided *t*-test comparing the average dilation between 500 and 3000 ms; df = 14, *t* = 4.32), and for one target than for no target (*p* < 0.05; df = 14, *t* = 1.84). This initial result is consistent with previous studies showing that pupil dilation is a sensitive measure of cognitive load (e.g., Hess and Polt, 1964).

To study how pupil size relates to target processing in the AB, the time course of the pupil size was aligned to the onset of T_1_ and averaged for each combination of lag and accuracy (Figure 2B). To study the difference in pupillary response between the one and two target conditions, the average pupil size in the single-target condition was subtracted from the two target conditions (Figure 2C).

Pupil dilation on no-blink trials (where both targets were correctly identified) was significantly larger than on blinked trials (where T_1_ was correctly identified but T_2_ was missed; *p* < 0.05, Monte–Carlo simulations; Figure 2C). A trace of dual-task interference was even observed when the analysis was restricted to trials with both targets correctly reported, as the peak amplitude of the pupil on no-blink trials was significantly smaller for lags 2 and 3 than for lags 6 and 7 (*p* < 0.05, Monte–Carlo permutation test).

In the PRP, a bottleneck is observed by which RT2 only proceeds after T_1_ completion, denting strict serial processing (Pashler, 1994; Sigman and Dehaene, 2005). This results in a classic profile where RT2 is insensitive to lag at the short lags (typically below ∼400 ms) but then starts “ramping” with a slope close to one. We examined whether the pupil can index this temporal dependency. Contrary to the PRP or to mixed PRP-AB experiments, this is a hidden physiological variable since in the AB subject’s report of T_1_ and T_2_ are delayed. We compared the pupil response to targets that differed by one lag, either inside (lag 2 vs. 3) or outside (lags 6 vs. 7) the interference period. No significant difference was observed between the pupillary response when comparing lags 2 and 3 (*p* = 0.76, permutation test). In contrast, the pupillary response was significantly different between lags 6 and 7 (*p* < 0.05, permutation test). To test whether the pupil dilation was delayed at short lags, we computed the first time for which the pupil dilation on no-blink trials reached 90% of its peak value, computed independently for each lag. Relative to the onset of T_2_, the pupil reached this level significantly later at shorter lags [Figure 2D; the slope of the latency was significantly higher than zero outside the interference range (lags 5–7, *p* < 0.05, permutation test), but not different from zero for the shorter lags (lags 1–3, *p* = 0.41)]. This result provides a physiological correlate of a delayed stage in the AB, as has been shown with EEG and fMRI (Sigman and Dehaene, 2008; Marti et al., 2011). Furthermore, contrary to the prediction of the majority of Attentional Blink models (Bowman and Wyble, 2007; Dux and Marois, 2009), a delay was also observed at lag-1, where T_1_ is immediately followed by T_2_. While this result argues against common explanations of lag-1 sparing which posit that both targets are processed in a single attentional event (Hommel and Akyurek, 2005), we did observe that the peak amplitude of the pupil was significantly larger for lag-1 than for lags 2 or 3 (*p* < 0.05 for both comparisons, permutation test).

Wierda et al. (2012) independently conducted an analysis very similar to ours in both aims and general conclusions. Besides analyzing the raw pupillary dilation, Wierda and colleagues adopted a method from fMRI research which consisted in deconvolving the pupillary response with an impulse response function to recover the timing of the attentional “pulses” that drive the physiological response. We applied this novel deconvolution technique to our pupillary data. The details of the method are as in Wierda et al. (2012) and should be sought in the original publication. The T_1_-locked pupillary response was assumed to result from the convolution of a set of attentional pulses time-locked to the visual stimuli and an empirically derived pupillary response function (Hoeks and Levelt, 1993; Wierda et al., 2012). Twenty pulses separated by the inter-stimulus interval (94.1 ms) were modeled, where the fourth pulse was aligned with T_1_. The strengths of the pulses were fitted to minimize the mean square error between the predicted and the observed pupil dilation. The strengths were calculated independently for each combination of participant, lag and accuracy. At long lags, the deconvolution technique identified two clearly distinct attentional events (Figure 2E right). While the first one was not affected by lag, the second one was locked to the onset of T_2_ (*p* < 0.05; permutation test comparing the center of mass of the pulse strength distributions for times between 500 and 1000 ms revealed later dilation for lag 7 than for lag 6). To test whether the relation between lag and pupillary response is lost at short lags, as observed in the analysis of the raw pupillary data, we compared the centers of mass of the pulse strength distributions for lags 1–3 over a temporal window of 0–500 ms, which revealed no significant difference (*p* > 0.25 for every pair-wise comparison between lags 1–3, permutation test). These results are consistent with the view that the pupil dilation tracks the timing of attention and reflects its limitations at short temporal scales.

Wierda and colleagues found that T_1_ activation was larger on blink trials than on no-blink trials. In our data, the absence of T_2_-locked components at the shortest lags makes it difficult to separate T_1_ and T_2_ processing. Evaluated over a temporal window of 200–500 ms from T_1_ onset, the strength of the pulses for lags 2 and 3 were significantly larger for no-blink trials (*p* < 0.005, permutation test comparing the average pulse strength on blink and no-blink trials; Figure 2E left). However, over a shorter time window of between 0 and 200 ms we did observe that the strength of the attentional pulses were larger on blinked trials (Figure 2E left; *p* < 0.05, permutation test), supporting the results of Wierda et al. (2012).

## Discussion

We investigated the dynamics of pupil dilatation in the Attentional Blink, a classic task used to study the limits of attention in time (Raymond et al., 1992; Vogel et al., 1998), to show that the pupil can be used to index events of behavioral relevance in the short time scale of ∼100 ms. We found that the pupil dilation on blinked trials was smaller than on no-blink trials. Dilation was also reduced for lags where the AB occurred even when T_2_ was correctly reported, showing a trace of interference even in the absence of a behavioral manifestation. Pupil dilation was not attenuated when T_2_ was immediately followed by T_1_, with amplitude that was comparable to that outside the interference, indicating that the attenuation was not due to an intrinsic limitation of the pupillary response. Inside the interference, the pupil dilation was protracted in time mimicking the pattern of response times observed in the PRP paradigm. The PRP and the AB are deeply related phenomena which can even be obtained within the same paradigm (Wong, 2002). At the behavioral level, the response time to T_1_ is known to modulate the duration of the AB effect (Jolicoeur, 1998, 1999). At the neuronal level, blinked and PRP trials produce similar patterns of brain activation which diverge for late frontal activations (Marti et al., 2011). Taatgen et al. (2009) showed that an ACT-R based model of multitasking originally developed for the PRP can explain multiple observations of the AB. Our results show a direct physiological correlate of delayed attentional selection in the AB consistent with the interpretation that the PRP and the AB tap the same processing limitations (Sigman and Dehaene, 2008; Zylberberg et al., 2010; Marti et al., 2011).

Lag-1 sparing has been difficult to account for by capacity-limit theories of the blink as it breaks the monotonic relation between lag and interference. Current theories of lag-1 sparing posit that when both targets are presented without intertwined distractors they are processed in a single cognitive episode (Di Lollo et al., 2005; Olivers and Meeter, 2008; Taatgen et al., 2009; Wyble et al., 2009), which can even encompass more than two uninterrupted targets (Di Lollo et al., 2005). Our finding of a delayed pupillary response suggests that some aspects of T_2_ processing are still postponed even at lag-1. This observation helps unify the AB and the PRP, as interference in the PRP is maximal when T_1_ and T_2_ are the closest. Delayed T_2_ consolidation does not imply that T_1_ and T_2_ are not processed simultaneously to some degree, as this is well established from behavioral experiments which have found robust interactions like reversals in the perceived order of targets and reduced accuracy for T_1_ (Hommel and Akyurek, 2005). Accordingly, we found that pupil dilation was larger at lag-1 than at lags 2 and 3, implying that attention is partially allocated to T_2_ while T_1_ is being processed (Zylberberg et al., 2009). Supporting this interpretation, a recent study combining the PRP and the “classification images” technique showed that T_2_ processing is not all-or-none but can partially proceed in parallel with T_1_, albeit with a reduced efficacy (Zylberberg et al., 2012).

The fine temporal resolution of the pupillary response is in line with a recent study by Wierda et al. (2012). The emphasis of their study was mainly on the methodological side, showing that the time course of the pupil dilation can be deconvolved to reveal the timing of attention. Our study is complementary since we investigate several lags within the AB (in the Wierda et al., 2012 study only one lag in the AB regime was investigated). This allowed us to investigate how the delay in the pupil response varies both within and outside of the interference regime, examining theories of central processing. Their deconvolution method applied to our data was consistent with our analysis of the raw pupillary dilation, showing that the relation between lag and dilation is maintained at long lags but is lost inside the interference period. Furthermore, higher pulse strength for T_1_ was found for blinked trials, confirming the results of Wierda et al. (2012) and highlighting the importance of T_1_ processing demands in modulating the AB (Jolicoeur, 1999).

## Materials and Methods

Fifteen subjects aged between 18 and 30 participated in the study. All had normal or corrected-to-normal vision, and were naive to the purpose of the experiment. Each subject performed 300 trials, in six blocks of equal number of trials. In each trial, a sequential stream of 19 items was presented in the center of a computer display (Figure 1A). Each item lasted 94.1 ms with no blanks in between. Subject had to report the letters embedded within the sequence of distracting numbers. The first target (T_1_) was always at position 4, 5, or 6, and could either be the only target in the sequence or be followed by a second target (T_2_) at a lag of 1–7 relative to the position of T_1_. We also included a condition where no targets were presented. Each subject performed 12 trials of each of the 25 conditions (21 dual-target, 3 single-target, 1 no-target). Targets were selected from the following list: “ABCDEFHJKPRTUV,” and distractors were numbers from 1 to 9. Items were green on a gray background. After the RVSP, subjects had to fixate a central dot before being allowed to make a response. Trials where subjects failed to maintain fixation or blinked were excluded from the analysis of pupil size. Gaze and pupil size were monitored with an Eyelink 1000 system at a sampling rate of 1000 Hz. Responses were made with the keyboard. Participants had to report the targets they had seen and then press the space bar to start the next trial.

Unless otherwise noted, the significance of the peak, latency, and pulse strength difference between two conditions (say A and B) was evaluated using a Monte–Carlo bootstrapping procedure (Roelfsema et al., 2003, their supplementary materials). We defined two simulated conditions, A′ and B′, and assigned the pupil dilation or pulse strength of subject *i* under condition A randomly to A′ or B′, and that of condition B to the remaining simulated condition. This random assignment was repeated for all subjects. Next, the average of the relevant statistic (peak dilation, latency, center of mass, amplitude) was computed for each conditions A′ and B′ and the difference between them was calculated. The procedure was repeated 20,000 times and the significance of the actually observed difference was determined by comparing it to this distribution.

Pupil deconvolution was implemented with the method and software provided by Wierda et al. (2012), with the same parameters as in their study unless otherwise noted.

## Conflict of Interest Statement

The authors declare that the research was conducted in the absence of any commercial or financial relationships that could be construed as a potential conflict of interest.
